# Sudden Cardiac Arrest in a Youth with Multiple Arrhythmic Substrates

**DOI:** 10.1155/2024/6054468

**Published:** 2024-04-08

**Authors:** James Ainsworth, Adrian Ionescu

**Affiliations:** Morriston Hospital, Morriston, Swansea, UK

## Abstract

**Background:**

Mitral valve prolapse (MVP) is a common condition with an estimated prevalence of 1-3%, in which there is systolic displacement of a morphologically redundant mitral valve towards the left atrium. Mitral annular disjunction (MAD) is a separation of the MV attachment with the left ventricle, with hypermobility of the leaflets, and with systolic “curling” of the basal LV (left ventricle) myocardium. It is frequently associated with MVP and may confer an increased arrhythmic risk. *Case Description*. A 28-year-old male had ventricular fibrillation leading to out-of-hospital cardiac arrest, which was successfully resuscitated. His coronary arteries were unobstructed on invasive coronary angiography. Transthoracic echocardiogram (TTE) demonstrated MAD, confirmed by cardiac magnetic resonance (CMR) imaging and transoesophageal echocardiogram (TOE). The LV was severely dilated with reduced EF (ejection fraction), and the QTc interval was also prolonged. His father had died suddenly aged 50 years.

**Conclusions:**

This report describes the clinical dilemma of identifying and treating a patient with multiple potential causes of cardiac arrest. Despite being relatively common, the clinical significance of MAD is still uncertain and the extent to which it may be linked with complications such as ventricular arrhythmias and sudden cardiac death. MAD appears to confer an increased risk of ventricular arrhythmias, particularly when associated with MVP, particularly nonsustained VT.

## 1. Introduction

Mitral valve prolapse (MVP) is defined by abnormal systolic displacement of one or both mitral leaflets into the left atrium [1]. Two to 4% of cases of sudden cardiac death (SCD) in the young have MVP. The mechanism is unclear, and causative association is uncertain, given the high prevalence of MVP, estimated at around 1-3% in the general population [1].

The mechanism of ventricular arrhythmias in MVP is speculative but may be related to structural alterations associated with the syndrome, such as mitral annular disjunction (MAD) [2] or myocardial replacement fibrosis. MAD is the displacement by at least 5 mm away from the crest of the LV myocardium of the insertion of the posterior mitral valve leaflet [1–3]. Disjunction of the annulus causes its functional decoupling from the LV, which can lead to altered contraction dynamics and abnormal annular movement, with paradoxical systolic expansion and flattening [2, 4, 5].

MAD may be diagnosed by transthoracic echo (TTE), transoesophageal echo (TOE), or cardiac CT, but cardiac magnetic resonance imaging (CMR) has emerged as the gold standard [1].

## 2. Case Presentation

A 28-year-old male was admitted following a witnessed, out-of-hospital episode of spontaneous ventricular fibrillation (VF) leading to cardiac arrest. He received immediate bystander CPR (cardiopulmonary resuscitation) and a total of 7 DC (direct current) shocks before return of spontaneous circulation. Total downtime was 24 minutes. He was previously healthy and well, with no cardiac history and good physical fitness, and had not used alcohol or recreational drugs prior to presentation. His father had died suddenly aged 50 years.

**Figure 1:**
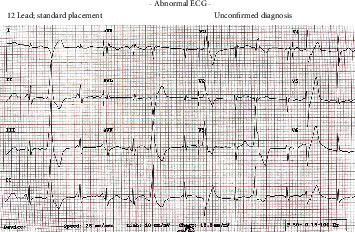
ECG from the day of admission to the hospital showing a long QTc and frequent ventricular ectopics.

**Figure 2:**
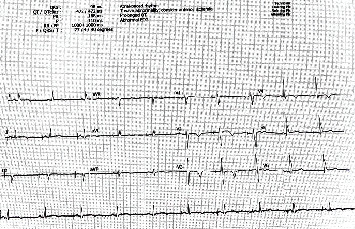
ECG from day 12 during admission following implantation of ICD showing persistent T-wave inversion.

**Figure 3:**
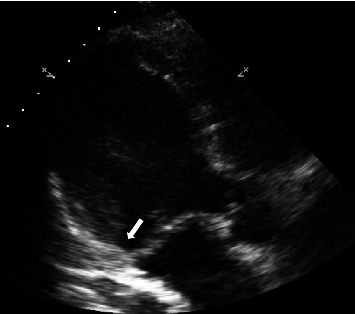
Systolic frame of an “off-piste” apical long-axis view demonstrating mitral annular disjunction (MAD)—arrow.

**Figure 4:**
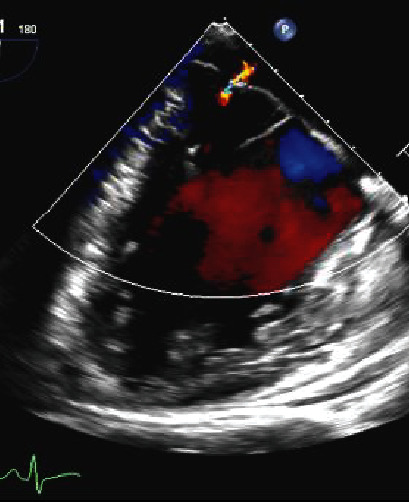
Transoesophageal systolic frame demonstrating MAD, as well as bileaflet MV prolapse and mild mitral regurgitation.

**Figure 5:**
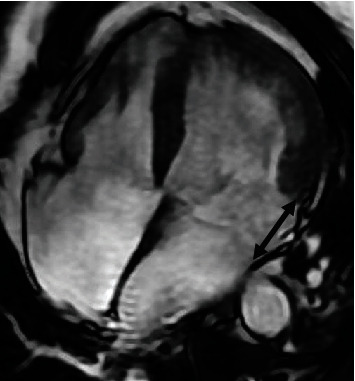
SSFP cardiac MRI image demonstrating a large gap between the insertion point of the posterior mitral leaflet and the top of the lateral wall myocardium—mitral annular disjunction (double-headed arrow).

The patient was extubated 24 h after admission and recovered fully, with initial confusion resolving within a few days. The first (3-lead) ECG (electrocardiogram) after ROSC (return of spontaneous circulation) demonstrated diffuse ST-segment depression and subsequent ECG monitoring documented multiple arrhythmias: sinus bradycardia, junctional escape rhythm, polymorphic ventricular ectopics (isolated and in self-terminating short runs), and prolonged QTc (472-511 ms). T wave inversion developed in V2-V4. Figures 1 and 2 show two patient ECGs from the day of admission and from day 12 during admission following implantation of ICD. Bedside TTE (see Figure 3), subsequently corroborated by TOE (see Figure 4) and by CMR (see Figure 5), demonstrated LV (left ventricular) dilatation and impairment, with LVEF (left ventricular systolic dysfunction) of 33% (Simpson's). There was MVP, with marked redundancy of the MV (mitral valve) leaflets, mild MR (mitral regurgitation), and MAD. There was no myocardial inflammation, infarction, infiltration, or fibrosis by CMR. The coronary arteries were unobstructed at invasive coronary angiography.

He received guideline-directed medical therapy (GDMT) for LV systolic dysfunction (spironolactone 25 mg once daily, bisoprolol 2.5 mg twice daily, ramipril 2.5 mg twice daily, and dapagliflozin 10 mg once daily), and 11 days after admission, an ICD (implantable cardioverter defibrillator) was implanted uneventfully; he remains well, under outpatient follow-up with the inherited cardiomyopathy team.

## 3. Discussion

We report a case of aborted SCD in a previously asymptomatic young adult with multiple substrates for VF, including the recently described MAD. There appears to be a significant association between MAD and MVP, itself, a common abnormality (estimated prevalence of 1-3% [1]), and both conditions have some association with SCD. Nevertheless, the significance of mitral annular disjunction remains uncertain, and with an estimated prevalence of 6-9%, it is increasingly recognised in subjects with structurally normal hearts, so it may be itself a normal anatomical variant [3, 5].

There is no clear quantitative threshold for the extent of mitral annular disjunction that confers an increased risk of SCD, making interpretation of this finding difficult. The incidence of nonsustained ventricular arrhythmias is higher with greater disjunction; one study reports that MAD > 8.5 mm may increase the risk of SCD up to sevenfold [3]. MAD without MVP is more common in young patients and may be associated with subsequent myxomatous degeneration and with the development of MVP [3].

A number of investigations may be carried out following a case of ventricular fibrillation or resuscitated sudden cardiac death, including history and examination, 12-lead ECG, laboratory investigations, and imaging techniques, including coronary angiography. Other tests may include exercise testing and Holter monitoring [6]. The diagnosis of MAD relies on the separation of the insertion of the mural leaflet into the left atrial wall and the base of the LV free wall of greater than or equal to 5 mm [1]. This may be diagnosed on TTE or TOE, but cardiac magnetic resonance imaging (CMR) is the gold standard for diagnosis [1].

Genetic testing may also have a role. MAD might have a genetic basis, leaving some individuals more prone to developing MAD ± MVP. There are many genes thought to be associated with abnormal mitral valve morphology. There is likely a complex overlapping genetic linkage between MVP and MAD, which is still largely unknown. The gene DCHS1 is an autosomal dominant gene which has also been linked with MAD in familial genetic studies. It has also been linked with MVP but may occur in the absence of MVP [7]. Genetic variants such as the DPP6 haplotype have been identified to have been linked with arrhythmias such as polymorphic ventricular tachycardia and idiopathic ventricular fibrillation, which are more common in patients with MVP and MAD [6]. A large number of other genes likely associated with MAD have also been identified, most of which however of uncertain significance [7].

There is no specific treatment guideline for MAD, and treatment approaches are usually followed according to management guidelines for valvular disease, heart failure, and arrhythmias, as specific management for arrhythmias associated with MVP or MAD is unclear [8]. Treatments may include medication, devices (implantable cardioverter defibrillator (ICD)), procedures (ablation), or surgical intervention [9]. In patients at risk of ventricular arrhythmias, the insertion of an ICD should be considered for secondary prevention [9], as in this case. Radiofrequency ablation may be done in some cases, such as those identified with frequent premature ventricular complexes on ECG or Holter monitor [6]. Surgical treatment is generally reserved for patients with MVP with significant mitral regurgitation, as there is no specific guideline for mitral valve surgery specifically for the treatment of ventricular arrhythmias in the absence of significant valve regurgitation [8].

Our patient's treatment was particularly difficult because he had multiple arrhythmic substrates; apart from MAD and MVP, there was severe LV dysfunction, as well as a prolonged QTC, and paternal history of SCD. The absence of genetic testing is a limitation of our presentation, which leaves a degree of uncertainty regarding the precise cause of cardiac arrest. The imaging studies defined a dilated cardiomyopathic phenotype, for which we could not identify a specific aetiology. There is a recognised association of MVP with LV dysfunction, but the mechanism remains speculative [10]. MVP and MAD may be associated with a dilated cardiomyopathy (DCM) phenotype [11] (with or without mid-wall myocardial fibrosis), which can be familial. Alternatively, an independent DCM may be present, as both DCM and MVP are common conditions. Ultimately, a pragmatic approach, the implant of an ICD, appeared to provide maximal protection.

## 4. Conclusion

Mitral valve prolapse with annular disjunction is currently emerging as a risk factor for sudden cardiac death, but its precise significance, pathogenesis, and treatment remain uncertain. Secondary prophylaxis with device implantation remains the essential treatment for patients with aborted sudden cardiac death, even when the exact substrate remains elusive.

## Data Availability

The data used to support the findings of this study are included within the article.
